# Assessing whether serum ceruloplasmin promotes non-alcoholic steatohepatitis via regulating iron metabolism

**DOI:** 10.5937/jomb0-37597

**Published:** 2023-01-20

**Authors:** Ziqiang Xia, Mei Hu, Liang Zheng, Endian Zheng, Min Deng, Jinming Wu, Xiong Sheng

**Affiliations:** 1 Wenzhou people's hospital, Department of Gastroenterology, Wenzhou, China; 2 The First Affiliated Hospital of Wenzhou Medical University, Department of Gastroenterology, Wenzhou, China; 3 The First Affiliated Hospital of Jiaxing College, Department of Infectious Diseases, Jiaxing, China; 4 The First Hospital of Jiaxing, Department of Infectious Diseases, Jiaxing, China

**Keywords:** ceruloplasmin, ferritin, non-alcoholic fatty liver disease, non-alcoholic steatohepatitis, ceruloplazmin, feritin, nealkoholna masna bolest jetre, nealkoholni steatohepatitis

## Abstract

**Background:**

Non-alcoholic steatohepatitis (NASH) is a progressive form of non-alcoholic fatty liver disease (NAFLD). The diagnostic gold standard for detecting NASH still relies upon an invasive pathological biopsy. There is, therefore, a need to identify non-invasive diagnostic markers. Oxidative stress mediates fatty liver progression to NASH. Imbalanced iron metabolism produces many reactive oxygen species (ROS). Ceruloplasmin is associated with oxidase and iron metabolism-related activities. The current study aimed to determine whether there was a correlation between ceruloplasmin levels and NASH and whether such a relationship may be associated with altered iron metabolism in NASH patients.

**Methods:**

A total of 135 NAFLD patients were enrolled in this study. A pathological biopsy confirmed that 60 of those patients had NAFLD activity scores (NAS) 5, while the remaining 75 had NAS<5.

**Results:**

Receiver operating characteristic (ROC) curves confirmed that serum ceruloplasmin and ferritin levels were predictors of NAS 5 and NAS<5, with area under the curve (AUC) values of 0.80 and 0.81, respectively. The serum ceruloplasmin levels in NAS 5 patients were significantly lower than those in NAS<5 patients (p< 0.001). Serum ceruloplasmin levels were also negatively correlated with ferritin levels. Lower serum ceruloplasmin levels were associated with more severe histopathological findings.

**Conclusions:**

Low serum ceruloplasmin and high serum ferritin are correlated with NASH. A high concentration of serum ferritin is a viable clinical biomarker of NASH, and low serum ceruloplasmin may participate in the occurrence of NASH by regulating iron load, which can be used as a non-invasive diagnostic marker of NASH.

## Abstract (sr)

**Uvod:**

Nealkoholičarski steatohepatitis (NASH) je progresivni oblik nealkoholne bolesti masne jetre (NAFLD). Dijagnostički zlatni standard za otkrivanje NASH se i dalje oslanja na invazivnu patološku biopsiju, te stoga postoji potreba da se pronađu neinvazivni dijagnostički markeri. Oksidativni stres posreduje u progresiji bolesti masne jetre u NASH. Neuravnotežen metabolizam gvožđa proizvodi mnoge reaktivne vrste kiseonika (ROS). Ceruloplazmin je povezan sa aktivnostima vezanim za oksidazu i metabolizam gvožđa. Ova studija je imala za cilj da utvrdi da li postoji korelacija između nivoa ceruloplazmina i NASH i da li takva veza može biti povezana sa izmenjenim metabolizmom gvožđa kod pacijenata sa NASH.

**Metode:**

U ovu studiju je bilo uključeno ukupno 135 pacijenata sa NAFLD. Patološka biopsija je potvrdila da je 60 od tih pacijenata imalo rezultate aktivnosti NAFLD (NAS) S 5, dok je preostalih 75 imalo NAS < 5.

**Rezultati:**

Krive radnih karakteristika prijemnika (ROC) su potvrdile da su nivoi ceruloplazmina i feritina u serumu bili prediktori za NAS S 5 i NAS < 5, sa vrednostima površine ispod krive (AUC) od 0,80 i 0,81, respektivno. Nivoi ceruloplazmina u serumu kod pacijenata sa NAS S 5 su bili značajno niži od onih kod pacijenata sa NAS < 5 (p < 0,001). Nivoi ceruloplazmina u serumu su takođe bili u negativnoj korelaciji sa nivoima feritina. Niži nivoi ceruloplazmina u serumu su bili povezani sa težim histopatološkim nalazima.

**Zaključak:**

Nizak serumski ceruloplazmin i visok serumski feritin su u korelaciji sa NASH. Visoka koncentracija serumskog feritina jeste klinički biomarker NASH, a nizak serumski ceruloplazmin može učestvovati u nastanku NASH regulacijom opterećenja gvožđem, što se može koristiti kao neinvazivni dijagnostički marker za NASH.

## Introduction

Non-alcoholic fatty liver disease (NAFLD) is the leading cause of chronic liver disease in most countries, particularly in Western countries [1]. NAFLD can gradually develop into non-alcoholic steatohepatitis (NASH), liver cirrhosis, and liver cancer [2], making it a significant research focus. The main characteristics of NASH are necrotizing inflammation caused by fatty liver infiltration. The main pathological features of NASH are steatosis, lobular inflammation, ballooning degeneration, and fibrosis [3]. NASH-mediated liver cirrhosis is projected to be the leading cause of liver transplantation by 2020 [4]. It is already the leading cause of liver transplantation in women [5]. Therefore, NASH's early diagnosis and treatment are crucial to prevent serious outcomes. At present, liver biopsy is the gold standard for NASH diagnosis. Still, the invasive nature of this diagnostic procedure and the associated risks preclude its widespread utilization. There is, therefore, a clear need to identify noninvasive diagnostic biomarkers of NASH.

Although the exact pathogenesis of NASH is still unknown, both the two-hit and the multiple-hit theories propose that oxidative stress is an important factor driving the occurrence of NASH [6] [7]. Dysfunction or dysregulation of proteins involved in metal metabolism and transport in vivo, including ceruloplasmin, ferritin, transferrin, and lactoferrin, can aggravate such oxidative stress [8]. Many studies have shown that high serum ferritin levels are associated with NASH [9] [10]. At the same time, ceruloplasmin regulates iron metabolism and oxidase activity. Ceruloplasmin, also known as copper oxidase, was first isolated from human serum by Holmberg & Laurell in 1948. It is a copper-containing α2 glycoprotein mainly produced in the liver that includes 6 copper-oxygenase domains of 150190 amino acids each [11] [12]. Serum ceruloplasmin can remove reactive oxygen species (ROS) and has anti-inflammatory activity. It serves as an acute response protein, and its levels increase twice or tri times in the context of inflammatory diseases [13]. Previous studies have found that ceruloplasmin is associated with Alzheimer's disease, which is itself closely linked to oxidative stress [14]. In addition, ceruloplasmin is also associated with cardiovascular diseases such as rheumatoid arthritis [15] and heart failure [16].

In this study, we examined serum ceruloplasmin levels in patients with NAFLD. We then used clinical laboratory data and liver histological findings to determine whether serum ceruloplasmin levels could be used as a non-invasive diagnostic marker of NASH. Moreover, we investigated whether serum ceruloplasmin levels were associated with the pathogenesis of NASH, either via regulating iron levels or by serving directly as an oxidase.

## Materials and methods

### Patients

The Ethics Committee approved the study of the First Affiliated Hospital of Wenzhou Medical University (WZ201611HP2506; November 25,2016). Samples were collected from patients in the Department of Infection at the First Affiliated Hospital of Wenzhou Medical University between February 2017 and September 2018. A total of 135 cases of NAFLD confirmed by pathological biopsy were enrolled in this study, including 60 patients with NAS 5 and 75 patients with NAS<5. All patients' informed consent was obtained. Exclusion criteria included a daily alcohol intake of more than 20 g, a history of liver disease, viral hepatitis, autoimmune liver disease, sclerosing cholangitis, primary biliary cirrhosis, drug-induced liver disease, and Wilson disease, aceruloplasminemia, α-1 antitryptase disease, or hemochromatosis.

### Clinical and laboratory evaluation

The height and weight of patients were measured in the morning, with all subjects wearing light clothes and no shoes using an electronic scale with the nearest error of 0.1 kg. Standing height was measured using a wall-mounted stadiometer to the nearest error of 0.1 cm in the morning. Body mass index (BMI) was calculated according to the formula: BMI = weight in kilograms/(height in meters) [2]. After overnight fasting of 12 hours, venous blood was collected from patients the following morning. These measurements were performed in an inpatient ward. Measured laboratory parameters in patient blood samples were examined in the Clinical Laboratory of the First Affiliated Hospital of Wenzhou Medical University, including low-density lipoprotein cholesterol (LDL-c), high-density lipoprotein cholesterol (HDL-c), alanine aminotransferase (ALT), aspartate aminotransferase (AST), gamma-glutamyl transpeptidase (GGT), insulin, platelet count (PLT), prothrombin time activity (PTA), prothrombin time (PT), glucose, glycated hemoglobin, ferritin, Cu, cerulo plasmin, hemoglobin, homocysteine, visceral fat area (VFA), triglycerides, total cholesterol, total bilirubin, and hs-CRP. A homeostasis model assessment of insulin resistance was used: (HOMA-IR)=(insulin×glucose/6.965)/22.5, where insulin and glucose were expressed in pmol/L and mmol/L, respectively. The aspartate aminotransferase-to-Platelet Ratio Index (APRI) was determined as follows: APRI=AST/(ULN)÷PLT×100, where PLT was expressed in 10^9^/L.

### Histologic examination

All patients underwent ultrasound-guided percutaneous liver biopsy. Pathological specimens were embedded in paraffin and stained with hematoxylin and eosin (H&E), Masson's trichrome, and reticulin. Sample grading was conducted by a pathologist who was blinded to clinical and laboratory assessments. Liver biopsy samples were assessed using the NASH standards for clinical research [17]. Four markers were assessed for each patient, including steatosis, lobular inflammation, ballooning, and fibrosis. The NAS score was assigned based on the degree of steatosis (0-3), lobular inflammation (0-3), and ballooning (0-2), with a total possible score of 0-8. Kleiner et al. [17] found that most patients with a NAS score 5 were NASH patients. In this study, all patients were divided into two groups - NAS 5 and NAS<5 - with patients diagnosed with NASH with a NAS value 5.

### Statistical analysis

SPSS v20.0 was used for all statistical analyses. The Shapiro-Wilk test evaluated the normal distribution of the continuous variables. Measurement data of normal distribution were statistically described as medians and 25^th^/75^th^ percentiles. They were analyzed via Mann-Whitney U-test for intra-group comparison. Categorical variables were expressed as percentages and were analyzed via chi-squared tests for inter-group comparison. A multivariable logistic regression analysis was performed to identify variables independently associated with NASH for variables that differed significantly between groups. The effectiveness of diagnosis was evaluated based upon receiver operating characteristic (ROC) curves, which were constructed based on the sensitivity and 1 minus specificity of all possible cut-off points. The area under the curve (AUC) of these ROC curves was used to express diagnostic accuracy, with AUC values closer to 1 indicating higher diagnostic accuracy. The best cut-off point for diagnosing NASH was when the sum of the sensitivity and specificity were maximized. Correlation coefficients were calculated via a Spearman correlation analysis. *P*<0.05 was considered statistically significant. 

## Results

### Patient characteristics

Table 1 compiles the results of liver biopsies from 135 patients (77 male and 58 female), including 60 patients with a NAS score 5 (33 male and 27 female) and 75 patients with a NAS score <5 (44 male and 31 female). Table 2 shows the demographic, clinical, and laboratory parameters for patients in these two groups. There were significant increases in BMI, ALT/AST, GGT, Homa-IR, APRI, ferritin, Hb, and TBIL in the NAS 5 group, relative to the NAS<5 group. In contrast, HDL-c, Cu, and CP levels were significantly lower in the NAS 5 group relative to the NAS<5 group. The multivariable logistic regression analysis results are shown in Table 3, revealing that ALT/AST, ferritin, and CP were all independently associated with NASH.

**Table 1:** Clinical and biochemical characteristics of patients with NAS 5 and NAS 5. Results are presented as medians (25th percentile, 75th percentile) for quantitative data and as percentages for qualitative data. The P values for quantitative data were calculated using the Mann-Whitney U-test. BMI, body mass index; LDL-c, low density lipoprotein-cholesterol, 2.07–3.10 mmol/L; HDL-c, high-density lipoprotein cholesterol, 1.16–1.42 mmol/L; ALT, alanine aminotransferase, 9–50 U/L; AST, aspartate aminotransferase, 15–40 U/L; GGT, gamma-glutamyl transpeptidase, 10–60 U/L; Insulin, 17.8–173 pmol/L; Homa-IR, homeostasis model assessment of insulin resistance; PLT, platelet count, (125 350)×109/L; PTA, prothrombin time activity, 70–140%; PT, prothrombin time, 11.5–14.6 second; APRI, Aspartate aminotransferase-to-Platelet Ratio Index; Glc, glucose, 3.9–6.1 mmol/L; GHb, glycated hemoglobin, 4.2–6.2%; ferritin, 23.9–336.2 Ug/L; Cu, 11.0–22.0 Umol/L; Cp, Ceruloplasmin, 20–60 mg/dL; Hb, hemoglobin,130–175 g/L; Hcy, Homocysteine, 0–15 Umol/L; VFA, visceral fat area; TG, triglyceride, 0.4–1.7 mmol/L; TC, total cholesterol, 2.44–5.17 mmol/L; TBIL, total bilirubin, 0–20 Umol/L; hs-CRP, 0–1mg/dL.

Variable	All (N=135)	NAS 5 (N=60)	NAS 5 (N=75)	P value
Sex (male)	77 (57.0%)	33 (55%)	44 (58.7%)	0.669
Age (years)	43(33–52)	41.5 (31.5–50.75)	44 (35–55)	0.274
BMI (kg/m^2^)	26.7 (24.25–28.73)	27.3 (25.7–29.3)	26 (23.7–28.1)	0.010
LDL-c (mmol/L)	2.95 (2.2–3.69)	3.14 (2.17–3.81)	2.83 (2.24–3.6)	0.601
HDL-c (mmol/L)	1 (0.89–1.12)	0.94 (0.81–1.04)	1.03 (0.93–1.21)	0.001
ALT (U/L)	54 (32–104)	90 (55–144.5)	37 (25–59)	0.001
AST (U/L)	35 (26–56)	52.5 (33.25–72.5)	29 (23–39)	0.001
ALT/AST	1.5 (1.2–2)	1.9 (1.5–2.2)	1.3 (0.9–1.6)	0.001
GGT (U/L)	51 (30–89)	67.5 (36.5–99)	40 (25–71)	0.001
Insulin (pmol/L)	103.7 (70.9–149.9)	118.8 (85.68–164.85)	89.7 (54.3–141)	0.003
Homa-IR	3.59 (2.32–5.23)	3.92 (2.61–5.6)	3.08 (2–4.98)	0.014
PLT (×10^9^/L)	237 (200–274)	230 (183.5–266.75)	245 (208–282)	0.052
PTA (%)	103 (97–113)	100.5 (97–112.75)	106 (97–113)	0.289
PT (second)	12.9 (12.3–13.2)	12.9 (12.33–13.38)	12.7 (12.3–13.2)	0.463
APRI	0.4 (0.3–0.6)	0.55 (0.4–0.8)	0.3 (0.2–0.4)	0.001
Glc (mmol/L)	5.1 (4.7–6.1)	5 (4.7–5.85)	5.25 (4.7–6.63)	0.277
GHb (%)	5.5 (5.3–6.1)	5.45 (5.23–6)	5.6 (5.3–6.5)	0.170
Ferritin (g/L)	247 (148.6–419.5)	381.6 (252.08–548.58)	168.7 (101.4–281.2)	0.001
Cu (mmol/L)	15.1 (14–16.1)	14.05 (13–15)	16 (15–18)	0.001
Cp (mg/dL)	22 (20–25)	21 (20–22)	24 (22–26)	0.001
Hb (g/L)	153 (139–161)	157.5 (152.5–167)	145 (130–155)	0.001
Hcy (Umol/L)	12 (10–13)	12 (11–13)	11 (10–14)	0.431
Body fat (kg)	21.2 (17.28–25.13)	22.1 (18.95–25.73)	19.75 (15.6–23.5)	0.024
VFA (cm^2^)	102.1 (88–121.9)	107.35 (92–125.63)	100.8 (86.4–118.4)	0.106
TG (mmol/L)	1.7 (1.14–2.47)	2.12 (1.21–2.61)	1.62 (1.13–2.25)	0.160
TC (mmol/L)	4.82 (4.04–5.57)	4.85 (4.14–5.67)	4.75 (3.97–5.45)	0.586
TBIL (mmol/L)	13 (10–17)	14.5 (11.25–18.75)	12 (10–16)	0.011
hs-CRP (mg/dL)	0.99 (0.55–1.92)	0.99 (0.69–1.74)	0.91 (0.53–1.97)	0.840

**Table 2:** Patient histological characteristics. Results are presented as percentages. Score, grade, and NAFLD activity score (NAS) were determined according to Kleiner et al. [17].

Histological feature	All (N=135)	NAS 5 (N=60)	NAS<5 (N=75)
Steatosis grade			
5%–33%	50 (37%)	1 (2%)	49 (65%)
34%–66%	50 (37%)	29 (48%)	21 (28%)
>66%	35 (26%)	30 (50%)	5 (7%)
Lobular inflammation (20x necrosis counted)			
0	14 (10%)	0 (0%)	14 (19%)
<2	73 (54%)	19 (32%)	54 (72%)
2-4	43 (32%)	36 (60%)	7 (9%)
>4	5 (4%)	5 (8%)	0 (0%)
Ballooning			
None	31 (23%)	1 (2%)	30 (40%)
Mild	84 (62%)	40 (67%)	44 (59%)
More than mild	20 (15%)	19 (32%)	1 (1%)
Fibrosis stage			
None	56 (41%)	9 (15%)	47 (63%)
Mild to moderate, zone 3, perisinusoidal, or periportal only	51 (38%)	28 (47%)	23 (38%)
Zone 3 and periportal, any combination	25 (19%)	21 (35%)	4 (7%)
Bridging	3 (2%)	2 (3%)	1 (2%)
Cirrhosis	0(0%)	0(0%)	0(0%)

**Table 3:** Logistic regression analysis with NAS as the dependent variable and other indicators as independent variables.

Factor	β	SE	Wals	P-value	OR value	95%CI
ALT/AST	1.125	0.493	5.201	0.023	3.079	1.171–8.094
Ferritin	0.006	0.001	16.217	0.001	1.006	1.003–1.009
Ceruloplasmin	-0.474	0.118	16.050	0.001	0.623	0.494–0.785
Constant	6.789	2.664	6.495	0.011	887.825	

### The correlation between serum ceruloplasmin, NASH, and ferritin

**Figure 1:**
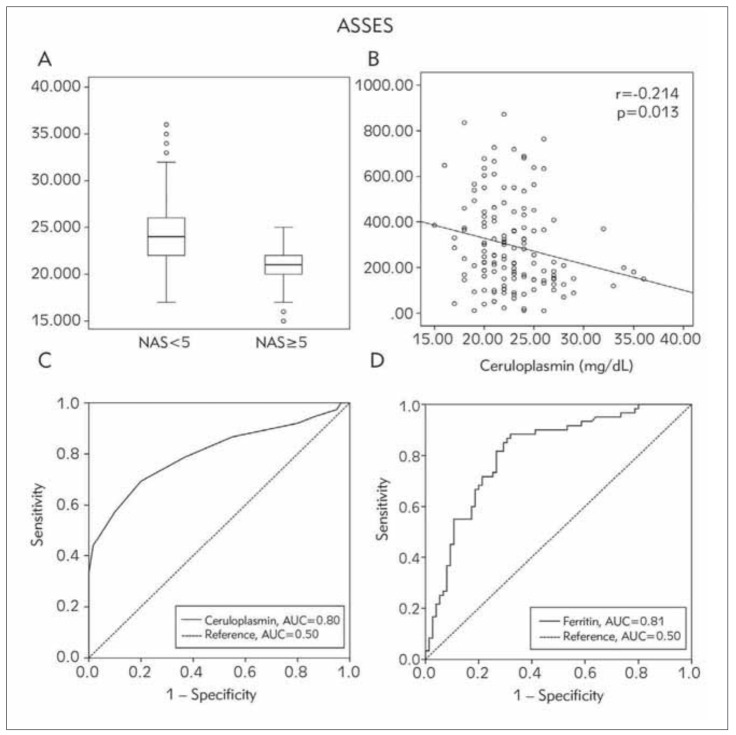
(A) The correlation between serum ceruloplasmin and NASH (r= 0.523, p<0.001). The box plots contain the values between the 25th and 75th percentiles. The thick line represents the median, and the thin lines correspond to the values range. Closed circles represent outliers. (B) The correlation between serum ceruloplasmin and ferritin (r=-0.214, p=0.013). Receiver operating characteristic (ROC) curves for serum ceruloplasmin (C) and ferritin (D) were used to discriminate between NA 5 and NAS 5. A cut-off ceruloplasmin value of 22.5 mg/dL predicted NAS 5 with a specificity of 0.8 and a sensitivity of 0.693. A cut-off ferritin value of 214.45 μg/L was predictive of NAS 5, with a specificity of 0.88 and a sensitivity of 0.68. AUC area under the curve.

As shown in (Figure 1a), lower ceruloplasmin levels were significantly associated with NASH (r = -0.523, *p*<0.001). To determine the relationship between serum ceruloplasmin and ferritin levels, we assessed the correlation between these two variables, revealing that they were significantly associated with one another (r = -0.214, *p*=0.013) (Figure 1b).

### Analysis of ROC curves for serum ceruloplasmin and ferritin

The ROC curves for serum ceruloplasmin and ferritin used to distinguish between the NAS 5 and NAS<5 groups are shown in Figure 1c and Figure 1d. The threshold serum ceruloplasmin value suitable for differentiating between patients with and without NASH was 22.5 mg/dL (*p*<0.001, 95% CI, 0.728-0.876). Using this threshold, the sensitivity and specificity values were 0.8 and 0.693, respectively (Figure 1c). The threshold value for serum ferritin was 214.45 μg/L (*p* < 0.001; 95% CI, 0.740-0.886), yielding to sensitivity and specificity values of 0.883 and 0.68, respectively (Figure 1d). The area under the ROC curve values for serum ceruloplasmin and ferritin levels were 0.80 and 0.81, respectively.

### The correlation between serum ceruloplasmin and liver histological findings

**Figure 2:**
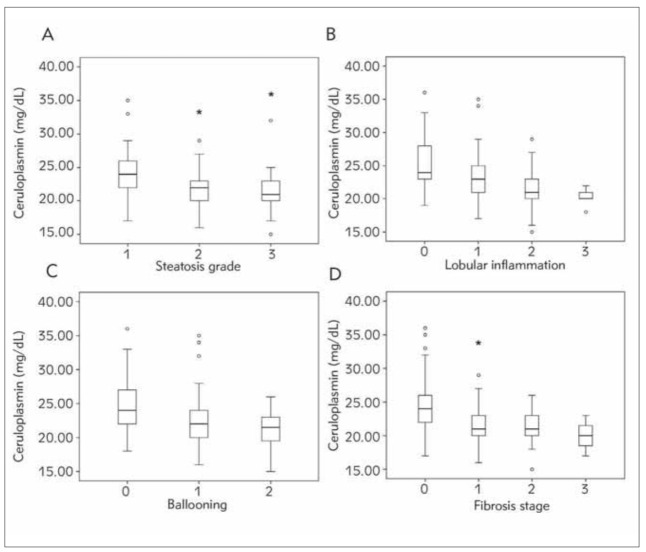
The correlation between serum ceruloplasmin and liver histological findings. The box plots contain the values between the 25th and 75th percentiles, with the thick line representing the median and the thin lines corresponding to the range of values. Closed circles represent outliers.

In order to further explore the significance of ceruloplasmin in NASH, we also studied patient liver histology findings. This approach revealed that serum ceruloplasmin was significantly correlated with steatosis (r = -0.35, *p*<0.001) (Figure 2a), lobular inflammation (r = 0.385, *p*<0.001) (Figure 2b), ballooning (r = -0.31, *p*<0.001) (Figure 2c), and fibrosis (r = -0.354, *p*<0.001) (Figure 2d).

## Discussion

This study found that serum ceruloplasmin levels in NAFLD patients with a NAS 5 were significantly lower than those in patients with a NAS<5. By assessing the AUC for ROC curves, we found that both serum ceruloplasmin and serum ferritin can be used to distinguish between patients with NAS values 5 and patients with NAS values <5.

There has been increasing interest in the relationship between iron load and NAFLD in recent years. Iron overload can facilitate the progression of NAFLD to NASH. Serum ferritin levels are the main indicator of iron storage *in vivo*. Kowdley et al. [10] found that serum ferritin is closely related to the severity of pathological conditions in patients with NAFLD and can be used for NASH diagnosis. Goh et al. [18] also detected a significant increase in serum ferritin levels in patients with NASH through a prospective cohort study. We also investigated the diagnostic value of serum ferritin and calculated the AUC of the ROC curve for this variable to be 0.81, indicating that a high concentration of serum ferritin is a viable clinical biomarker of NASH. Valenti et al. [19] also found that phlebotomy treatment can reduce the iron load in patients, thereby improving insulin resistance and reducing the degree of liver damage in those with NASH. Iron overload promotes the occurrence of NASH by activating the NF-B pathway in hepatic macrophages [20] [21], leading to ROS production. In the two-hit theory of NAFLD, the first "hit" is thought to be liver fat accumulation. The second is thought to be the damage caused by oxidative stress [6]. When iron overload occurs, large quantities of ROS are produced via the Fenton reaction of ROS are produced via the Fenton reaction (i.e. Fe^2+^+ H_2_O_2_ Fe^3+^+ (OH^-^) + OH , H_2_O_2_ + Fe^3+^ Fe^2+^+ O_2_ + 2 H^+^, O_2_ + Fe^3+^ Fe^2+^ + O_2_ ) [22]. Iron overload can produce a large number of oxygen free radicals, ROS, and hydroxyl free radicals, leading to hepatic cell damage. At the same time, iron overload can stimulate the activation of hepatic macrophages and the subsequent secretion of a wide range of inflammatory factors, further driving the development of NASH. However, other studies have found that most patients with NASH show no evidence of significant iron accumulation [23] [24]. Therefore, the role of liver iron load in the pathogenesis of NASH remains controversial and unresolved.

The mechanism leading to an increased iron index in patients with NASH remains unknown. Ceruloplasmin is an oxidase that participates in the body's metabolism of copper and iron. Ceruloplasmin can oxidize Fe^2+^ to Fe^3+^, and it is also able to stabilize transferrin on the cell membrane. Fe^3+^ then binds to transferrin, allowing its export from the liver [25]. Previous studies have found that molecular oxygen can increase the ceruloplasmin-mediated Fe^2+^ oxidation rate *in vitro*. It has been suggested that ferric oxidation is necessary for the formation of transferrin [26] [27]. In a study of copper-deficient pigs, it was found that copper deficiency impaired the ability of iron in hepatic parenchymal cells to be exported into the plasma, resulting in iron deposition in the liver [28]. Another study found that male patients with Wilson's disease exhibited low serum ceruloplasmin accompanied by high serum ferritin levels [29]. This is consistent with our finding that low serum ceruloplasmin levels were correlated with high serum ferritin levels. In summary, ceruloplasmin is an iron oxidase. As such, a lack of ceruloplasmin can drive the accumulation of Fe^2+^, some of which may be absorbed by ferritin and converted into Fe^3+^ for storage, while the remaining iron may lead to NASH via the Fenton reaction, leading to extensive ROS production. We also examined the relationship between serum ceruloplasmin and NASH, revealing that NASH patients had significantly lower serum ceruloplasmin levels than those without this disease. An ROC curve analysis revealed that serum ceruloplasmin was able to differentiate NAFLD patients based on their NAS values (NAS 5 vs. NAS<5). This suggests that serum ceruloplasmin may participate in the occurrence of NASH by regulating iron load, and that serum ceruloplasmin levels can be used as a non-invasive diagnostic marker of NASH. As this study was based solely on clinical, laboratory and pathological data, the specific molecular mechanisms underlying these observations will still need to be assessed in future studies.

In conclusion, we found that decreased ceruloplasmin levels and increased ferritin levels are correlated with NASH. Patient ceruloplasmin levels can be used to help distinguish between patients based on the NAS score (NAS 5 vs. NAS<5). They can thus be used as a non-invasive diagnostic marker of disease status. Ceruloplasmin levels are negatively correlated with ferritin levels. Therefore, we propose that serum ceruloplasmin mediates NASH by regulating iron metabolism. This study provides noninvasive diagnostic markers and clinical therapeutic target pathways for treating patients with NAFLD.

## Dodatak

### Acknowledgments

This study was funded by the Science and Technology Bureau of Jiaxing (Nos. 2018AD32078), Zhejiang Provincial Health Science and Technology Program (2021KY1108), Jiaxing City Medical Key Discipline - Infectious Diseases (2019-zc-02), Jiaxing Key Laboratory of Virus-related Infectious Diseases.

### Conflict of interest statement

All the authors declare that they have no conflict of interest in this work.
